# Cultural and neighborhood characteristics associated with activity-specific parenting practices in Hispanic/Latino youth: a secondary analysis of the Hispanic Community Children’s health study/study of Latino youth

**DOI:** 10.1007/s10865-023-00395-w

**Published:** 2023-02-02

**Authors:** Christopher J. Gonzalez, Madison N. LeCroy, Martha L. Daviglus, Linda Van Horn, Linda C. Gallo, Franklyn Gonzalez, Krista M. Perreira, Maria M. Llabre, Martin F. Shapiro, Carmen R. Isasi

**Affiliations:** 1 Division of General Internal Medicine, Department of Medicine, Weill Cornell Medical College, 420 E 70Th St., LH-300, New York, NY 10065 USA; 2 Department of Epidemiology and Population Health, Albert Einstein College of Medicine, 1300 Morris Park Avenue, Bronx, NY 10461 USA; 3 Institute for Minority Health Research, University of Illinois at Chicago, 1819 W. Polk Street, Suite 246 (M/C 764), Chicago, IL 60612 USA; 4 Department of Preventive Medicine, Feinberg School of Medicine, Northwestern University, 580 North Lake Shore Drive, Chicago, IL 60611 USA; 5 Department of Psychology, San Diego State University, 5500 Campanile Drive, San Diego, CA 92182 USA; 6 Department of Biostatistics, University of North Carolina at Chapel Hill, 123 W. Franklin St., CB# 8030, Chapel Hill, NC 27516 USA; 7 Department of Social Medicine, School of Medicine, University of North Carolina at Chapel Hill, 333 South Columbia Street, CB #7240, Chapel Hill, NC 27599 USA; 8 Department of Psychology, University of Miami, P.O. Box 248185, Coral Gables, FL 33124 USA

**Keywords:** Social determinants, Physical activity, Sedentary behavior, Parenting practices, Hispanic/Latino

## Abstract

Hispanic/Latino youth are less physically active than non-Hispanic/Latino youth. We assessed whether activity-specific parenting practices relate to moderate-to-vigorous physical activity (MVPA) and sedentary behavior among Hispanic/Latino youth, and whether cultural (acculturation) and neighborhood characteristics (perceived barriers to activity) relate to the use of parenting practice patterns. Using the Hispanic Community Children’s Health Study/Study of Latino Youth (SOL Youth, *n* = 976 8–16-year-olds), we modeled linear regression associations between parenting practices and mean daily MVPA and sedentary behavior. Parenting practice patterns were then developed using k-means cluster analysis, and regressed on parental acculturation and neighborhood characteristics. Discipline predicted higher MVPA in females (β 1.89 [95% CI 0.11–3.67]), while Monitoring/Reinforcement predicted higher MVPA in males (β 4.71 [95% CI 0.68–8.74]). Three patterns were then identified: Negative Reinforcement (high Limit Setting and Discipline use), Positive Reinforcement (high Limit Setting and Monitoring/Reinforcement use), and Permissive Parenting (low parenting practice use). Higher acculturation predicted use of Positive Reinforcement. Activity-specific parenting practices are associated with activity in sex-specific ways among Hispanic/Latino youth, and cultural factors predict the use of parenting practices.

**Supplementary Information:**

The online version contains supplementary material available at 10.1007/s10865-023-00395-w.

## Background

Childhood obesity disproportionately impacts Hispanic/Latino youth 2 to 19 years old in the United States (US), with the prevalence nearly twice that of non-Hispanic/Latino White youth of that age (26% vs.14%) (Skinner et al., 2018). This has substantial implications for the long term cardiovascular health of the largest ethnic minority population in the US (Burke et al., 2008; Center, 2015; Isasi et al., 2016; K. Liu et al., 2012; Serdula et al., 1993). While increased moderate-to-vigorous physical activity (MVPA) and limited sedentary behavior have been associated with favorable health and weight outcomes among children and adolescents (Carson et al., 2016; Janssen & LeBlanc, 2010; Strizich et al., 2018; Tremblay et al., 2011) Hispanic/Latino children 6 to 11 years old are less likely to meet national physical activity recommendations than non-Hispanic/Latino White children (OR 0.60 [95% CI 0.38–0.95]), (Fakhouri et al., 2013) and Hispanic/Latino adolescents 11 to 16 years old report lower levels of physical activity overall than their non-Hispanic/Latino White counterparts (Iannotti & Wang, 2013).

Parents and caregivers are often strong influences on the behaviors of children (Birch & Fisher, 1998, 2000). Various food and activity-specific parenting practices, defined as content-specific parental actions that may intentionally or unintentionally influence the nutritional intake and physical activity of children, have previously been associated with children’s weight status (Birch et al., 2001; Lloyd et al., 2014; Shloim et al., 2015). Numerous studies have assessed the role of parenting practices on food-specific obesity-promoting behaviors, but there is limited evidence exploring their role on physical activity and sedentary behaviors (Vaughn et al., 2016; Yee et al., 2017). While the operationalization of activity-specific parenting practices has varied, practices that encourage physical activity, through actions such as co-participation in activity or providing transportation to facilitate physical activity, have generally been associated with more physical activity (Dowda et al., 2011; Schary et al., 2012). However, the role of other parenting practices, such as setting rules and limits on sedentary behavior (including time spent watching television) or simply monitoring (i.e. keeping track of) sedentary behavior, are less established, with studies often limited by small and relatively younger samples of pre-school children (Hutchens & Lee, 2017; Xu et al., 2015). Moreover, despite significant racial and ethnic inequities in physical activity and sedentary behavior among youth, existing studies on activity-specific parenting practices have lacked diverse samples, and few studies have explored their influence on the physical activity of Hispanic/Latino youth specifically. A recent review assessing the role of activity-specific parenting practices among Hispanic/Latino youth found only 16 studies published in over 20 years, and the authors concluded that existing studies were limited by samples lacking diversity in Hispanic/Latino background and notable heterogeneity in measures of parenting practices (Lindsay et al., 2018).

Prior evidence suggests that parenting practices may differ by race and ethnicity (Chao & Kanatsu, 2008; Roche et al., 2007). Hispanics/Latinos often face unique challenges that may impact their health behaviors, including physical activity, but also potentially impact activity-specific parenting practices (Lindsay et al., 2018; Velasco-Mondragon et al., 2016). Acculturation, the process by which immigrants adopt beliefs, values, and behaviors from a new host culture while potentially maintaining those of the culture of origin, has been associated with increased physical activity among Hispanic/Latino youth (Afable-Munsuz et al., 2010; J. Liu et al., 2009). Acculturation and acculturative stress, i.e. stress related to acculturation such as perceived discrimination or language conflicts, have been associated with the use of obesity-promoting food-specific parenting practices, but their associations with activity-specific parenting practices have not been delineated (LeCroy et al., 2019; Power et al., 2015). Activity-specific parenting practices may also vary across neighborhoods: in a small sample of pre-school children from various neighborhoods of a Texas city, perceived neighborhood traffic safety and availability of places to be physically active were positively associated with activity-specific parenting practices that encourage physical activity among Hispanic/Latino youth, while perceived traffic hazards and stranger danger were associated with practices that discourage physical activity (O’Connor et al., 2014). Neighborhood socioeconomic factors such as less crime, higher safety, and higher social order have been associated with favorable physical activity and weight outcomes, (Franzini et al., 2009; Molnar et al., 2004) but their role on activity-specific parenting practices among diverse Hispanic/Latino youth remains to be assessed.

To better understand the role of activity-specific parenting practices among a broader Hispanic/Latino population and among varying cultural and neighborhood contexts, we studied whether certain activity-specific parenting practices were associated with higher levels of physical activity and lower levels of sedentary behavior among Hispanic/Latino youth aged 8–16 years old. Specifically, we assessed whether Limit Setting (setting limits on undesired behaviors) was associated with lower physical activity and higher sedentary activity, and whether Reinforcement (approving of desired behaviors) was associated with higher physical activity and lower sedentary activity. Additionally, we assessed whether higher levels of parent acculturation to US culture and fewer perceived neighborhood barriers to activity were associated with more effective activity-specific parenting practice patterns. Notably, to study activity-specific parenting practices, we exploratorily aimed to derive and confirm a new factor structure for the activity-specific parenting practice items in the Parenting strategies for Eating and Activity Scale (PEAS).

## Methods

### Study population

The Hispanic Community Children’s Health Study/Study of Latino Youth (SOL Youth) is a cross-sectional, ancillary study of the Hispanic Community Health Study/Study of Latinos (HCHS/SOL) (Isasi et al., 2014). HCHS/SOL is a cohort study of 16,415 self-identified Hispanic/Latino adults (ages 18–74 years) who were selected using a stratified, two-stage probability sampling design across four US communities (Bronx, New York; Chicago, Illinois; Miami, Florida; San Diego, California) (Lavange et al., 2010; Sorlie et al., 2010). The present study examines one of the three main objectives of SOL Youth: to examine the associations of parenting practices with youth’s lifestyle behaviors, including physical activity and sedentary behavior. SOL Youth was conducted between 2012 and 2014, during which the study enrolled 1466 children aged 8 to 16 years living in the household of a parent/caregiver (referred to as the parent throughout this manuscript) who completed the HCHS/SOL baseline examination. This corresponded to 1020 parents from HCHS/SOL. SOL Youth study participation included three components: (1) an initial clinical examination at the field center, (2) 7 days of wearing a physical activity monitor, and (3) a second 24-h dietary recall via telephone within a month of the initial clinic visit. Protocols for HCHS/SOL and SOL Youth are published elsewhere (Isasi et al., 2014; Lavange et al., 2010; Sorlie et al., 2010).

### Activity-specific parenting practices

Parents reported their activity-specific parenting practices using the Parenting strategies for Eating and Activity Scale (Larios et al., 2009). PEAS is a 26-item questionnaire that measures five parenting practices related to diet (16 items) and physical activity (10-items): Limit Setting (setting limits on undesired behaviors), Discipline (correcting undesired behaviors), Control (incite desired behavior), Monitoring (tracking behaviors), and Reinforcement (approving of desired behaviors) (Birch et al., 2001; Larios et al., 2009). Parents responded to each item using a 5-point Likert scale (1 = disagree to 5 = agree or 1 = never to 5 = always, depending on the parenting practice item).

PEAS was developed over a decade ago to assess food- and activity-specific parenting practices among Hispanic/Latino parents and has been utilized in a variety of studies assessing parenting practices and the obesogenic behaviors of Hispanic/Latino youth, including SOL Youth (Lindsay et al., 2018). However, the original factor structure was validated among Hispanic/Latino mothers of 5- to 8-year-olds and assessed food- and activity-specific parenting practices jointly (Larios et al., 2009). There remains a need to evaluate whether this widely used measure can be applied to assessing activity-specific parenting practices and their association with physical activity and sedentary behavior among a broader age group of Hispanic/Latino youth. Thus, as a secondary outcome, we derived and confirmed a new factor structure to measure only the activity-specific parenting practices in PEAS (see Statistical Analysis).

### Physical activity and sedentary behavior

Frequency, duration, and intensity of physical activity was assessed using the ActiCal™ (MiniMiter Respironics®, Bend, OR) accelerometer (model 198-0200-03) worn above the iliac crest on the right hip for 7 days. Accelerations were captured every 15 s, and non-wear time was defined as consecutive zero counts for at least 90 min, allowing for short time intervals with nonzero counts lasting up to 2 min if no counts were detected during both the 30 min upstream and downstream from that interval (Choi et al., 2011). Physical activity thresholds for MVPA and sedentary behavior were based on prior calibration studies of the Actical accelerometer, which subsequently established cut points used to assess MVPA in SOL Youth: sedentary behavior 0–17 counts counts/15-s; light activity, 18–440 counts/15-s; moderate activity, 441–872 counts/15-s; and vigorous activity ≥ 873 counts/15-s (Evenson et al., 2019; Gallo et al., 2017; Romanzini et al., 2014; Strizich et al., 2018). As in prior studies, and to facilitate practical interpretation, MVPA and sedentary behavior were summed and average minutes/day calculated (Evenson et al., 2019). Adherence to accelerometer protocol was defined as ≥ 8 h of wear time for at least three days (Evenson et al., 2019).

### Barriers to neighborhood activity and acculturation

All questionnaires were interviewer-administered in the participant’s language of preference (English or Spanish). Mean scores for the derived variables were set to missing if individuals were missing responses for more than one item used to calculate the means.

#### Perceived barriers to neighborhood activity

Parents reported whether it is difficult to be active in the local park or street/neighborhood near their home using a 9-item questionnaire adapted from the Neighborhood Impact on Kids Study (Isasi et al., 2014; Tappe et al., 2013). The questionnaire had two subscales: perceived lack of appropriate play areas (6 items; Cronbach’s α = 0.81) and perceived crime (3 items; Cronbach’s α = 0.63). The questionnaire prompt was, “It is difficult to be active in the local park or streets/neighborhood near our home because…”, and example items from each subscale include, “there is no equipment (basketball hoop, etc.)” (lack of appropriate play areas subscale) and, “it’s not safe because of crime (strangers, gangs, drugs)” (perceived crime subscale). Participants responded using a 4-point Likert scale and received a mean score for each of the two subscales, with higher scores indicating greater perceived barriers to activity.

#### Neighborhood socioeconomic status

Parents reported neighborhood crime and social issues using a 5-item questionnaire adapted from the Children of Immigrants Longitudinal Study (Isasi et al., 2014; Portes & Rumbaut, 2007). An example item is, “How much of a problem in your neighborhood is the following: drug use or drug dealing out in the open”. Parents responded to each item using a 3-point Likert scale and received an overall mean score, with higher scores indicating more neighborhood issues (Cronbach’s α = 0.88).

#### Acculturation

Parents reported their acculturation using the 12-item Brief Acculturation Rating Scale for Mexican Americans-II (Brief ARSMA-II) (Bauman, 2005). The Brief ARSMA-II primarily assesses language use and preference using 5-point Likert responses for two subscales: the 6-item Anglo Orientation Scale (AOS; Cronbach’s α = 0.86) and the 6-item Hispanic/Latino Orientation Scale (LOS; Cronbach’s α = 0.82). Example items from each subscale include, “I enjoy English language movies” (AOS) and, “My thinking is done in the Spanish language” (LOS). Parents received an average score for each subscale (LeCroy et al., 2019).

#### Acculturative stress

Parents reported stress related to acculturation using a 9-item Acculturative stress index about experiences with discrimination, language, and intergenerational differences (Gil et al., 1994). An example item includes, “How often has it been hard for you to get along with others because you don’t speak English well?”. This index has been validated and used in numerous studies of acculturative stress, including prior assessments of the HCHS/SOL sample used in our study (Gil et al., 1994; LeCroy et al., 2021).

### Covariates

#### Anthropometrics

Trained examiners measured child and parent’s weight and height (in triplicate) using a standardized protocol. Body mass index (BMI) was subsequently calculated for each participant in kg/m^2^.

#### Socio-demographics

Children reported their age and sex. Parents reported their own age and sex, highest education, household income, Hispanic/Latino background, and years living in the US (i.e. born in US 50 states or the District of Columbia, foreign-born living in the US ≥ 20 years, or foreign-born living in the US < 20 years.

### Statistical analysis

To identify the activity-specific parenting practices measured by PEAS, an exploratory factor analysis of the ten activity-specific PEAS items was conducted on a random split half-sample of the parents with complete PEAS data (*n* = 501). This was followed by a confirmatory factor analysis in the remaining validation half-sample (*n* = 501) (van Prooijen & van der Kloot, 2001). Factors were retained by the proportion criterion and were rotated using varimax rotation (Birch et al., 2001; LeCroy et al., 2019). One item (“I offer television, videos, or video games to my children as a reward for good behavior”) that loaded < 0.30 on all factors was removed, and the exploratory factor analysis was re-run (Osborne & Banjanovic, 2016). Factors were labeled according to the variables with factor loadings ≥|0.30|, using names in accordance with the original PEAS factor structure (Larios et al., 2009). Individuals received mean scores for each identified factor within a potential range of 1 to 5, with higher scores indicating more use of a parenting practice.

For regression models, individuals were excluded if they had missing accelerometer data (n = 358) or missing data on any covariate (n = 132). The final analytic sample size included 976 youth. There were no significant differences among variables used in our analysis between individuals who were excluded versus included. To examine the association between the identified activity-specific parenting practices and average MVPA and sedentary behavior, separate multivariable linear regression models were used. All identified activity-specific parenting practices were simultaneously included in each model because parenting patterns generally do not occur in isolation. As a sensitivity analysis, models were re-run using the original PEAS factor structure instead of the derived parenting practices.

**Fig. 1:**
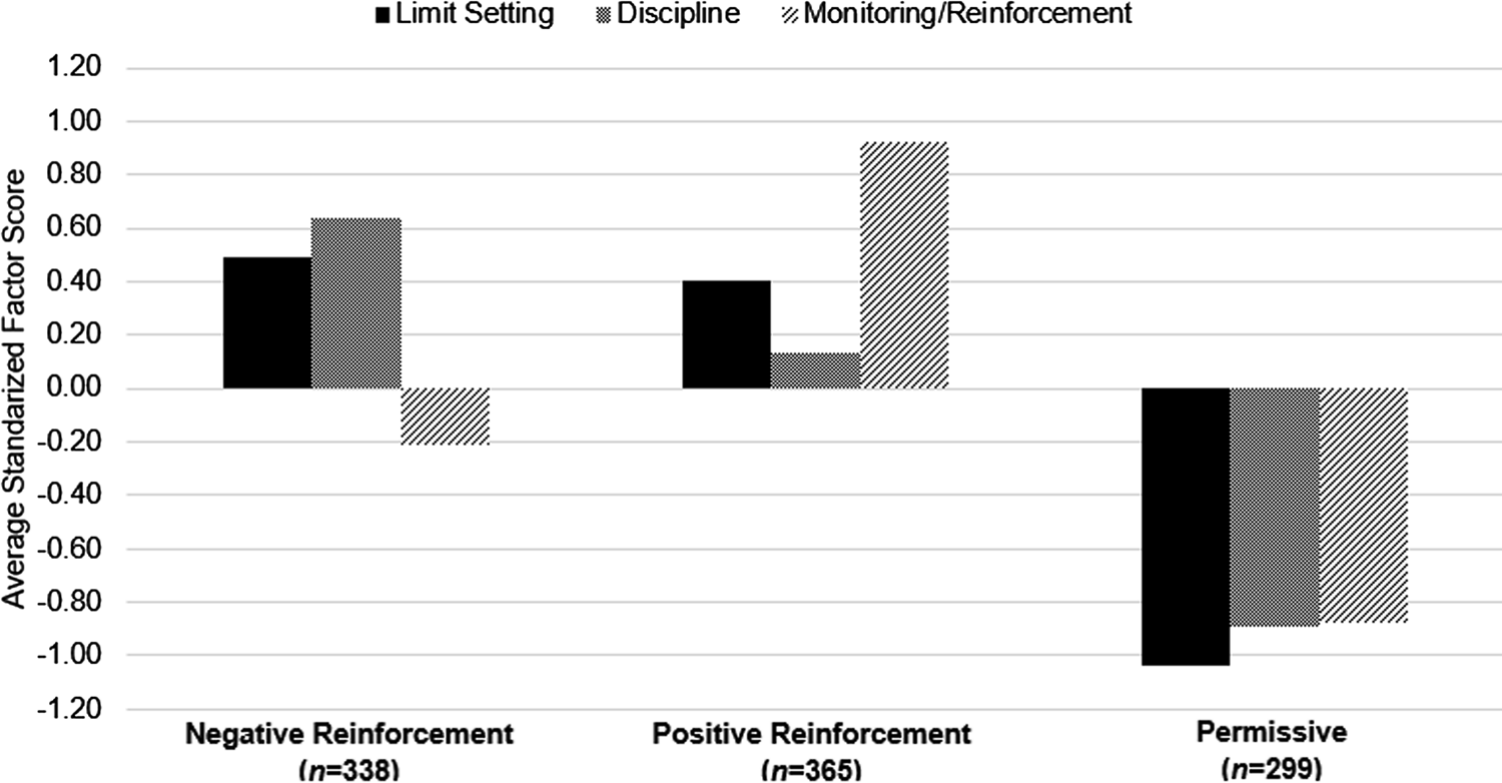
Clusters derived from k-means cluster analysis based on standardized factor scores in SOL Youth parents (*n* = 1002)

Because parents generally do not enact individual parenting practices separately, we then derived activity-specific parenting practice patterns using a k-means cluster analysis based on z-transformed scores for the mean factor scores. For the k-means cluster analysis, cluster solutions with 2 to 10 clusters were examined. Each analysis was run for a maximum of 1000 iterations, and seeds containing less than or equal to 5% of the sample were removed during each iteration (Everitt et al., 2011). The best solution was selected according to the pseudo-F statistic and is presented in Fig. 1 (Caliński & Harabasz, 1974; Milligan & Cooper, 1985).

To examine the association of parent-perceived neighborhood characteristics (perceived lack of appropriate play areas; perceived crime; and neighborhood socioeconomic status) and acculturation-related measures (years parent lived in the US, parent’s AOS and LOS scores, and parent’s acculturative stress) with the use of activity-specific parenting practice patterns, we used multinomial regression models using derived parenting practice patterns as the outcome.

All aforementioned models included child’s age, parent’s age, child’s sex, parent’s sex, parent’s education, household income, child’s BMI, parent’s BMI, parent’s Hispanic/Latino background, and SOL Youth field center. Child’s age group and sex were examined as potential effect modifiers in all regression models by including interaction terms between these binary variables and the main exposures of interest. Presence of effect modification was based on a *p*-value < 0.10 for the interaction term. All regression analyses and descriptive statistics accounted for stratification and for clustering by primary sampling units and were weighted to adjust for sampling probability of selection and nonresponse with the use of complex survey procedures in SAS software version 9.4 (SAS Institute).

## Results

A description of the target population of SOL Youth is provided in Table 1. Approximately half of the population was male (50.2%) with a mean age of 12 years. Families were predominately low-income (54.7% reported household income < $20,000), and about half of parents had lived in the US for < 20 years. On average, youth participated in MVPA for 36.0 min/day and were sedentary for 608.3 min/day (~ 10.1 h/day).

**Table 1:** Characteristics of the target population of SOL Youth (*n* = 976)

	Mean or n (SE or %)
*Socio-demographics*	
Child’s sex (*n* and %)	
Female	492 (49.8)
Male	484 (50.2)
Parent’s sex (*n* and %)	
Female	835 (88.8)
Male	141 (11.2)
Child’s age, years (mean and SE)	12.0 (0.1)
Parent’s age, years (mean and SE)	41.1 (0.3)
Child’s BMI, kg/m^2^ (mean and SE)	22.3 (0.2)
Child’s BMI percentile group (*n* and %)*	
Underweight	28 (3.1)
Normal	465 (49.6)
Overweight	214 (21.1)
Obesity	170 (16.2)
Severe obesity	99 (9.9)
Parent’s BMI, kg/m^2^ (mean and SE)	30.8 (0.4)
Parent’s education (*n* and %)	
< High school	366 (37.3)
High school or equivalent	275 (29.3)
> High school	335 (33.4)
Household income (*n* and %)	
< $20,000	527 (54.7)
$20,000–$40,000	303 (29.1)
> $40,000	146 (16.2)
Parent’s Hispanic/Latino background (*n* and %)	
Central American	92 (7.6)
Cuban	87 (7.1)
Dominican	116 (14.1)
Mexican	474 (50.6)
Puerto Rican	108 (11.8)
South American	72 (6.1)
Mixed/Other	27 (2.7)
*Acculturation*	
Years parent lived in US (*n* and %)	
Born in US	126 (13.2)
< 20 years	476 (49.5)
≥ 20 years	374 (37.2)
Parent’s ARSMA-II Brief (mean and SE; range: 1–5)	
Anglo orientation scale	2.7 (0.1)
Latino orientation scale	4.2 (0.05)
*Parent’s acculturative stress* (mean and SE; range: 1–5)	1.8 (0.04)
*Parent’s perceived barriers to child’s physical activity*	
Appropriate play areas (mean and SE; range: 1–4)	2.1 (0.04)
Crime (mean and SE; range: 1–4)	1.8 (0.04)
*Neighborhood socioeconomic status* (mean and SE; range: 1–3)	1.6 (0.03)
*Outcome*	
Average moderate to vigorous activity, minutes/day (mean and SE)^†^	36.0 (1.1)
Average sedentary behavior, min/day (mean and SE)^‡^	608.3 (5.2)

Unweighted *n* (weighted %)

SD, standard deviation; BMI, body mass index; US, United States; ARSMA-II Brief, Acculturation Rating Scale for Mexican Americans-II Brief

*Underweight: BMI < 5th percentile; Normal: BMI 5^th^–84th percentile; Overweight: BMI 85th–94th percentile; Obesity: BMI > 95th percentile, 125% of 95th percentile and BMI < 35; Severe obesity: BMI > 95th percentile, 125% of 95th percentile or BMI > 35

^†^Moderate to vigorous physical activity defined as ≥ 441 counts/15 s, reported in minutes/day

^‡^Sedentary behavior defined as ≤ 17 counts/15 s, reported in minutes/day

Three parenting practices were identified (Table 2) and were named Limit Setting, Discipline, and Monitoring/Reinforcement. The Limit Setting and Discipline factors matched the original PEAS structure exactly. Confirmatory factor analysis results showed this factor structure had X^2^ = 282.26, df = 24; Root Mean Square Error of Approximation [RMSEA] = 0.15 [90% CI 0.13, 0.16]; Standardized Root Mean Square Residual [SRMR] = 0.07; and Bentler’s Comparative Fit Index [CFI] = 0.84. SRMR < 0.08 indicated a good model fit, with the three-factor solution reproducing the data well (Hu & Bentler, 1999). Bentler’s CFI was less than the model fit criteria of ≥ 0.95, suggesting low average correlation between variables (Hu & Bentler, 1999; Wang & Wang, 2019). RMSEA was greater than the model fit criteria of < 0.10, potentially due to the limited number of model parameters or small sample size (Kenny et al., 2014).

**Table 2:** Exploratory factor analysis of physical activity-specific parenting practices (*n* = 501)*

Parenting item	Limit Setting	Discipline	Monitoring/Reinforcement
I limit the amount of time my children play video games (like Game Boy, Sega, PlayStation) or are on the computer during the weekend (Sat/Sun)	**0.71**	0.13	0.03
I limit the amount of time my children watch TV or videos during the weekend (Sat/Sun)	**0.68**	0.09	0.14
I limit the amount of time my children play video games (like Game Boy, Sega, PlayStation) or are on the computer during the week (Mon-Fri)	**0.58**	0.20	0.24
I limit the amount of time my children watch TV or videos during the week (Mon-Fri)	**0.53**	0.21	0.37
I often discipline my children for watching TV or videos without my permission	0.18	**0.76**	0.20
I often discipline my children for playing video games of the computer without my permission	0.20	**0.76**	0.20
I keep track of the exercise my children are getting	0.16	0.09	**0.61**
I keep track of the amount of TV or videos my children are watching	0.36	0.20	**0.53**
I often praise my children for being physically active	0.04	0.14	**0.47**

Factor loadings above 0.40 are given in bold

Associations between the identified activity-specific parenting practices and MVPA and sedentary behavior were null for the overall sample (Supplementary Table 1). However, exploratory analyses indicated that child’s sex modified associations of Discipline and of Monitoring/Reinforcement with MVPA (Table 3). Specifically, among male youth, a 1-point increase in the parent’s score for Monitoring/Reinforcement was associated with youth participating in 4.71 (95% CI 0.68, 8.74) additional minutes of MVPA. Among female youth, a 1-point increase in the parent’s score for Discipline was associated with youth participating in 1.89 (95% CI 0.11, 3.67) additional minutes of MVPA. Though the interaction term for Discipline and age group was significant in the model for MVPA (*p*-for-interaction = 0.04), the stratified results showed no evidence of effect modification by child’s age group for this or any other associations (Supplementary Table 2).

**Table 3:** Associations of physical activity-specific parenting practices with average MVPA and sedentary behavior (minutes/day), stratified by child’s sex (*n* = 976)

	p-for-int	*β coefficients (95% CI)*	
		Male (*n* = 484)	Female (*n* = 492)
*MVPA*^†^			
Limit Setting	0.65	1.24 (− 1.91, 4.40)	0.66 (− 1.23, 2.56)
Discipline	0.06*	− 0.65 (− 2.70, 1.40)	1.89 (0.11, 3.67)*
Monitoring/Reinforcement	0.004*	4.71 (0.68, 8.74)*	− 1.48 (− 4.06, 1.10)
*Sedentary behavior*^‡^			
Limit Setting	0.22	5.11 (− 5.49, 15.71)	− 3.75 (− 12.06, 4.57)
Discipline	0.86	− 2.39 (− 11.54, 6.76)	− 1.42 (− 9.86, 7.01)
Monitoring/Reinforcement	0.28	− 11.69 (− 27.88, 4.50)	− 1.74 (− 12.93, 9.45)

*p < 0.05

*MVPA* moderate to vigorous physical activity; *p-for-int* p-value for interaction term

^†^Moderate to vigorous physical activity defined as ≥ 441 counts/15 s, reported in minutes/day

^‡^Sedentary behavior defined as ≤ 17 counts/15 s, reported in minutes/day

All models are survey-weighted and adjusted for the following covariates: child’s age, parent’s age, child’s sex, child’s sex*parenting practice, parent’s sex, child’s BMI, parent’s BMI, parent’s education, household income, SOL Youth center, Hispanic/Latino background

As a sensitivity analysis, associations of parenting practices with MVPA and sedentary behavior were also explored using the original PEAS factor structure (Supplementary Tables 3–5). Findings are similar to those reported using the derived parenting practices, with greater Monitoring being associated with higher MVPA (and lower sedentary behavior) in males only, and greater Discipline being associated with higher MVPA in females only.

K-means cluster analysis revealed three parenting practice patterns (Fig. 1): Negative Reinforcement (high scores for Limit Setting and Discipline), Positive Reinforcement (high scores for Limit Setting and Monitoring/Reinforcement) and Permissive (low scores for all parenting practices). Table 4 shows associations of each neighborhood characteristic and acculturation-related measure with parenting practice cluster membership (Permissive cluster membership as the referent group). Neighborhood characteristics did not relate to cluster membership, but one acculturation-related measure, AOS score, significantly related to Positive Reinforcement. Specifically, a one-point increase on the AOS subscale of the Brief ARSMA-II was associated with a 1.59 (95% CI 1.06, 2.40) times greater odds of belonging to the Positive Reinforcement compared to Permissive cluster.

**Table 4:** Associations of physical activity-specific parenting practice pattern clusters with neighborhood and acculturation measures (*n* = 976)

	Overall F-test, *p*-value	Odds Ratios (95% CI)	
		Negative Reinforcement	Positive Reinforcement
Perceived barriers, appropriate play areas	0.10	1.36 (0.90, 2.06)	1.58 (1.04, 2.39)
Perceived barriers, crime	0.36	0.91 (0.58, 1.44)	0.72 (0.45, 1.15)
Neighborhood SES	0.38	1.43 (0.77, 2.66)	1.48 (0.83, 2.64)
Parent acculturative stress	0.68	0.89 (0.57, 1.37)	0.80 (0.49, 1.31)
*Parent acculturation*			
AOS	0.03*	1.04 (0.65, 1.66)	1.59 (1.06, 2.40)*
LOS	0.46	0.79 (0.49, 1.23)	0.98 (0.62, 1.55)
*Parent nativity*	0.13		
US-born		0.44 (0.15, 1.31)	0.26 (0.08, 0.82)*
Lived in US ≥ 20 years		0.65 (0.33, 1.28)	0.43 (0.20, 0.91)*

*p < *0.05*

*SES* socioeconomic status, *AOS* Anglo-Orientation Scale, *LOS* Latino Orientation Scale

Model is survey-weighted and adjusted for the following covariates: child’s age, parent’s age, child’s sex, parent’s sex, child’s BMI, parent’s BMI, parent’s education, household income, SOL Youth center, Hispanic/Latino background, years parent lived in US, ARSMA-II Brief, and acculturative stress

Exploration of effect modification indicated that the association between AOS score and membership in the Positive Reinforcement cluster may be modified by child’s age (*p*-for-interaction = 0.10) and sex (*p*-for interaction = 0.07; data not shown in table). Among youth aged 12–16 years, a one-point increase on the AOS subscale was associated with a 1.86 (95% CI 1.10, 3.16) times greater odds of belonging to the Positive Reinforcement compared to Permissive cluster. Additionally, among males only, a one-point increase on the AOS subscale was associated with a 2.31 (95% CI 1.37, 3.90) times greater odds of belonging to the Positive Reinforcement compared to Permissive cluster.

## Discussion

This study assessed whether activity-specific parenting practices were associated with physical activity and sedentary behavior in Hispanic/Latino youth, and whether cultural and neighborhood environmental factors were associated with the use of more effective activity-specific parenting practice patterns among Hispanic/Latino parents. Activity-specific parenting practices were not associated with either MVPA or sedentary behavior among the entire sample of Hispanic youth. However, Discipline was associated with higher MVPA in females only, and Monitoring/Reinforcement was associated with higher MVPA in males only. Finally, except for Anglo-oriented acculturation, cultural and perceived neighborhood characteristics were not associated with the use of any activity-specific parenting practice patterns.

The associations between parenting practices and activity-specific behaviors have been inconsistent in existing literature (Lindsay et al., 2018). Our findings suggest that this lack of consistency in prior research may be due to studies measuring parenting practices too broadly and reveal that activity-specific parenting practices are associated with physical activity in sex-specific ways. Our analyses showed that higher levels of Discipline were associated with higher MVPA in females, but not in males, while higher levels of Monitoring/Reinforcement were associated with higher MVPA in males, but not in females. While it is possible that males and females respond differently to parenting practices, there is no evidence to suggest that a child’s sex would impact their response to a certain parenting practice, nor that Discipline is more effective among females while Reinforcement is more effective among males. Instead, these associations may have emerged from differences in baseline physical activity behaviors, as well as differences in the ways parents use activity-specific parenting practices. For example, parents may execute parenting practices differently based on the sex of the child, using different forms of Discipline and/or Monitoring/Reinforcement for males than for females, and vice versa.

The PEAS measure assesses when Discipline is used (i.e., “for watching television without permission”), but it does not assess the methods of Discipline used. As such, there may be sex-based variability on the methods of Discipline used and their subsequent effectiveness. A recent meta-analysis of sex-associated parenting methods suggests that there is little sex-related difference in the parenting strategies used among males and females; however, this meta-analysis was underpowered to assess these differences, particularly as it relates to physical discipline (Endendijk et al., 2016)**.** Prior evidence suggests that parents are indeed more likely to use harsh discipline and physical control strategies in boys relative to girls, particularly in Western countries and among parents with strong attitudes about gender-stereotypes (Endendijk et al., 2017; Meier et al., 2009; Taillieu et al., 2014). Within this context, and considering that physical discipline has been found to be ineffective, (Gámez-Guadix et al., 2010; Mulvaney & Mebert, 2007) the sex-specific associations observed between Discipline and MVPA in our study may reflect differences in the specific type of Discipline used among Hispanic/Latino males and females.

Alternatively, traditional gender norms may differentially cue parents as to when to implement parenting practices in males and females. Prior studies have reported that boys are more likely than girls to receive parental support when involved in team sports and active chores, while girls are more likely to receive support for mild-activity chores, particularly among Hispanics/Latinos (Anderson et al., 2009; Cong et al., 2012; Mendonça et al., 2015). This observation was attributed to potential differences in what parents believe are gender-appropriate activities for children, particularly in the context of traditional Hispanic/Latino cultural gender norms. Gender-specific perceptions regarding activity may contribute to parents monitoring or reinforcing different kinds of physical activities for males and females, so that reinforcement of activity is unlikely to be associated with higher MVPA in the latter, as was seen in our study. Future research should aim to assess sex-related differences in the behaviors encouraged or discouraged by certain activity-specific parenting practices, and potentially tailor interventions to redirect parenting practices that encourage MVPA rather than less strenuous physical activity. Notably, activity-specific parenting practices were not associated with sedentary behavior in our analyses, including those assessing sex or age as potential modifiers. This may reflect limtiations in the influence or measure of activity-specific parenting practices, though studies assessing their association with sedentary behaviors have been limited, and the results inconsistent (Gubbels et al., 2011; Lindsay et al., 2018; McGuire et al., 2002).

Additionally, we found that Hispanic/Latino parents with relatively higher Anglo-orientation scores were more likely to use a parenting practice pattern that included Monitoring/Reinforcement, while higher Latino-orientation scores, perceived neighborhood barriers to activity and perceived neighborhood socioeconomic status did not relate to parenting practice patterns. Though prior literature suggests that physical activity in youth increases with acculturation, acculturation is a complex process and the factors contributing to these associations remain unclear (Allen et al., 2014; Williams et al., 2010)**.** Cultural differences in parenting practices have been qualitatively described in the past, and one quantitative study of Hispanic/Latino pre-school children found that cultural variables, including increased acculturation to US culture, were weakly associated with parents discouraging physical activity due to safety concerns (Calzada et al., 2012; O’Connor et al., 2014; Sherry et al., 2004). Our findings add to our understanding of how cultural variables are related to activity-specific parenting practices, and suggest acculturation is associated with certain patterns of parenting practices, particularly those that include Monitoring/Reinforcement and are less likely to include Discipline. The directionality of these associations warrants further evaluation.

Our study does not support prior reports that perceived characteristics of the neighborhood, such as safety or barriers to activity, may contribute to activity-specific parenting practices (O’Connor et al., 2014; Xu et al., 2015). Within a socio-ecological model of physical activity that considers potential social and environmental influences, and considering that Monitoring/Reinforcement was positively associated with MVPA among males in our sample, our findings suggest that parenting practices associated with acculturation may be one factor influencing the activity trends of Hispanic/Latino youth (Davison & Birch, 2001)**.** Future studies should aim to assess the pertinence of community-level factors not measured in our analyses.

Finally, while Hispanic/Latino youth are disproportionately burdened by obesity, and measuring the proximal factors associated with weight-related behaviors is critical to developing targeted interventions that address these inequities, few measures of activity-specific parenting practices have been validated in a diverse sample of Hispanic/Latino youth (Hales et al., 2017; Lindsay et al., 2018; Patrick et al., 2013). Our findings confirm that the activity-specific items in the PEAS, a measure used in multiple studies of Hispanic/Latino youth, are an acceptable measure of activity-specific parenting practices among Hispanic/Latino youth 8 to 16 years old (Larios et al., 2009; Mojica et al., 2019; Yin et al., 2019). While the factor structure that emerged in our factor analyses was generally consistent with the original factor structure of the PEAS questionnaire, the single activity-specific item representing the Reinforcement construct in the original PEAS loaded on to the same factor as the Monitoring items, and was included in our analyses, but the single activity-specific Control item in the original PEAS questionnaire was not included. The Control parenting practice has not been extensively studied as it relates to physical activity and sedentary behavior, but prior studies of dietary patterns suggest Control is a parenting practice frequently utilized among Hispanic/Latino parents, and that it is associated with certain obesogenic behaviors (Conlon et al., 2015; Loth et al., 2013; Wehrly et al., 2014). Future studies should consider further tailoring activity-specific parenting practice measures that assess Control and should also explore factors that potentially influence sedentary behaviors.

### Strength and limitations

This study used a rigorous exploratory and confirmatory factor analysis to validate the activity-specific parenting practice measure in a large sample of Hispanic/Latino youth across multiple geographic areas and a wide range of ages (8–16 years old). The study also assessed physical activity using accelerometers, allowing us to assess associations of parenting practices with objectively measured physical activity and sedentary behavior, thus addressing one of the key aims of SOL Youth. While the specifics of the planned analyses were not pre-registered, the proposed analysis (including requested variables and proposed models) underwent internal review with HCHS/SOL and SOL Youth prior to releasing data to the present authors (Isasi et al., 2014). The present study also had some limitations. The sample had a relatively low socioeconomic status overall and consisted of Hispanics/Latinos in four primarily urban sites, which may have limited our ability to distinguish within-group differences related to socioeconomic or environmental differences. There was considerable missingness in accelerometry data, and we are unable to assess whether MVPA and sedentary behaviors differed between those with and without accelerometry data. Additionally, accelerometer accuracy in measuring various forms of physical activity and sedentary behavior can vary considerably (Lynch et al., 2019). Finally, physical activity and sedentary behavior patterns among Hispanic youth may have changed from those observed in SOL Youth between 2012 and 2014.

## Conclusion

Activity-specific parenting practices are associated with moderate-to-vigorous activity in sex–specific ways but have no association with sedentary behavior in Hispanic/Latino youth. Among acculturation and neighborhood characteristics, only Anglo-orientation is related to activity-specific parenting patterns. More research is needed to understand how acculturation is influencing activity-specific parenting practices, and longitudinal studies are needed to assess if parenting practices can be leveraged to change Hispanic/Latino children’s activity.

### Supplementary Information

Below is the link to the electronic supplementary material.

Supplementary file1 (DOCX 21 kb)

## Data Availability

Due to the data restrictions imposed by the governing IRBs that oversee this human subject research, data access in HCHS/SOL is limited. Researchers can apply for access to the public use data sets for specific research projects by contacting the data curators at NHLBI. Interested researchers should visit the BIOLINCC page on the data set to learn how to obtain HCHS study data. Accession number HLB01141418a.

## Abbreviations

US: United States
MVPA: Moderate-to-vigorous physical activity
OR: Odds ratio
CI: Confidence interval
HCHS/SOL: Hispanic Community Health Study/Study of Latinos
SOL Youth: Hispanic Community Children’s Health Study/Study of Latino Youth
PEAS: Parenting strategies for Eating and Activity Scale
Brief ARSMA-II: Brief Acculturation Rating Scale for Mexican Americans-II
AOS: Anglo Orientation Scale
LOS: Hispanic/Latino Orientation Scale
BMI: Body mass index
RMSEA: Root mean square error of approximation
SRMR: Standardized root mean square residual
CFI: Comparative fit index
