# Supplementary material for: Cultural and neighborhood characteristics associated with activity-specific parenting practices in Hispanic/Latino youth: a secondary analysis of the Hispanic Community Children’s health study/study of Latino youth
Source: J Behav Med. 2023 Feb 2;46(5):732–44. doi: 10.1007/s10865-023-00395-w (PMC10558378; doi:10.1007/s10865-023-00395-w)
Supplement: Supplementary file 1 — Supplementary file1 (DOCX 21 kb) [file 10865_2023_395_MOESM1_ESM.docx]

**Supplementary Table 1.** Associations of physical activity-specific parenting practices with average MVPA (minutes/day) and sedentary behavior (minutes/day) (*n*=976)

| **Physical activity-specific parenting practice** | ***β coefficients (95% CI)*** | |
| --- | --- | --- |
|  | **MVPA*** | **Sedentary**^†^ |
| **Limit Setting** | 0.98 (-0.98, 2.94) | 0.13 (-6.94, 7.19) |
| **Discipline** | 0.23 (-1.35, 1.82) | -1.07 (-8.18, 6.03) |
| **Monitor/Reinforce** | 1.65 (-0.92, 4.22) | -5.89 (-17.15, 5.37) |

*MVPA* moderate to vigorous physical activity

All models are survey-weighted and adjusted for the following covariates: child’s age, parent’s age, child’s sex, parent’s sex, child’s BMI, parent’s BMI, parent’s education, household income, SOL Youth center, Hispanic/Latino background

*Moderate to vigorous physical activity defined as ≥441 counts/15 seconds, reported in minutes/day

^†^Sedentary behavior defined as ≤17 counts/15 seconds, reported in minutes/day

**Supplementary Table 2.** Associations of physical activity-specific parenting practices with average MVPA and sedentary behavior (minutes/day), stratified by child’s age group (*n*=976)^*^

|  | **p-for-int.** | ***β coefficients (95% CI)*** | |
| --- | --- | --- | --- |
|  |  | **Young** (*n*=458) | **Old** (*n*=518) |
| **MVPA**^†^ |  |  |  |
| Limit Setting | 0.29 | 2.24 (-0.33, 4.81) | 0.68 (-1.68, 3.05) |
| Discipline | 0.04 | -0.94 (-2.78, 0.91) | 1.36 (-0.52, 3.25) |
| Monitoring/Reinforcement | 0.69 | 1.60 (-1.92, 5.12) | 0.91 (-1.58, 3.40) |
| **Sedentary behavior**^‡^ |  |  |  |
| Limit Setting | 0.54 | 1.60 (-8.68, 11.89) | -1.59 (-11.49, 8.31) |
| Discipline | 0.47 | 1.88 (-6.62, 10.39) | -4.03 (-13.51, 5.45) |
| Monitoring/Reinforcement | 0.64 | -2.30 (-15.86, 11.27) | -8.41 (-21.54, 4.72) |

*MVPA* moderate to vigorous physical activity; *p-for-int.* p-value for interaction term

*Young = 8-11 years of age, Old = 12-16 years of age

^†^Moderate to vigorous physical activity defined as ≥441 counts/15 seconds, reported in minutes/day

^‡^Sedentary behavior defined as ≤17 counts/15 seconds, reported in minutes/day

All models are survey-weighted and adjusted for the following covariates: child’s age group, child’s age group*parenting practice, parent’s age, child’s sex, parent’s sex, child’s BMI, parent’s BMI, parent’s education, household income, SOL Youth center, Hispanic/Latino background

**Supplementary Table 3.** Associations of physical activity-specific parenting practices with average moderate to vigorous physical activity (minutes/day) and sedentary behavior (minutes/day), using the original PEAS factors (*n*=976)

| **Physical activity-specific parenting practice** | ***β coefficients (95% CI)*** | |
| --- | --- | --- |
|  | **MVPA*** | **Sedentary**^†^ |
| Limit Setting | 0.82 (-1.17, 2.81) | 0.60 (-6.38, 7.59) |
| Monitoring | 1.62 (-0.46, 3.69) | -7.85 (-16.46, 0.77) |
| Discipline | 0.17 (-1.46, 1.80) | -1.05 (-8.29, 6.19) |
| Control | 0.26 (-0.87, 1.40) | 1.11 (-3.56, 5.78) |
| Reinforcement | 0.01 (-1.56, 1.58) | 2.00 (-5.34, 9.35) |

*MVPA* moderate to vigorous physical activity

All models are survey-weighted and adjusted for the following covariates: child’s age, parent’s age, child’s sex, parent’s sex, child’s BMI, parent’s BMI, parent’s education, household income, SOL Youth center, Hispanic/Latino background

*Moderate to vigorous physical activity defined as ≥441 counts/15 seconds, reported in minutes/day

^†^Sedentary behavior defined as ≤17 counts/15 seconds, reported in minutes/day

**Supplementary Table 4.** Associations of physical activity-specific parenting practices with average MVPA and sedentary behavior (minutes/day), stratified by child’s sex, using the original PEAS factors (*n*=976)

|  | **p-for-int.** | ***β coefficients (95% CI)*** | |
| --- | --- | --- | --- |
|  |  | **Male** (*n*=484) | **Female** (*n*=492) |
| **MVPA**^†^ |  |  |  |
| Limit Setting | 0.69 | 1.02 (-2.24, 4.28) | 0.55 (-1.28, 2.39) |
| Monitoring | 0.02 | 3.82 (0.64, 7.00)* | -0.66 (-2.81, 1.49) |
| Discipline | 0.06 | -0.70 (-2.77, 1.38) | 1.81 (0.07, 3.56)* |
| Control | 0.87 | 0.26 (-1.43, 1.94) | 0.24 (-1.00, 1.47) |
| Reinforcement | 0.26 | 0.80 (-1.70, 3.30) | -0.79 (-2.41, 0.83) |
| **Sedentary behavior**^‡^ |  |  |  |
| Limit Setting | 0.11 | 6.88 (-3.98, 17.74) | -4.41 (-12.68, 3.86) |
| Monitoring | 0.07 | -15.83 (-29.01, -2.66)* | -0.85 (-10.61, 8.92) |
| Discipline | 0.74 | -1.99 (-11.07, 7.08) | -2.07 (-10.56, 6.42) |
| Control | 0.47 | 0.23 (-7.14, 7.59) | 2.52 (-2.60, 7.64) |
| Reinforcement | 0.49 | 4.49 (-6.29, 15.26) | -0.58 (-8.63, 7.47) |

*p<0.05

*MVPA* moderate to vigorous physical activity; *p-for-int.* p-value for interaction term

^†^Moderate to vigorous physical activity defined as ≥441 counts/15 seconds, reported in minutes/day

^‡^Sedentary behavior defined as ≤17 counts/15 seconds, reported in minutes/day

All models are survey-weighted and adjusted for the following covariates: child’s age, parent’s age, child’s sex, child’s sex*parenting practice, parent’s sex, child’s BMI, parent’s BMI, parent’s education, household income, SOL Youth center, Hispanic/Latino background

**Supplementary Table 5.** Associations of physical activity-specific parenting practices with average MVPA and sedentary behavior (minutes/day), stratified by child’s age group, using the original PEAS factors (*n*=976)^*^

|  | **p-for-int.** | ***Odds ratios (95% CI)*** | |
| --- | --- | --- | --- |
|  |  | **Young** (*n*=458) | **Old** (*n*=518) |
| **MVPA**^†^ |  |  |  |
| Limit Setting | 0.43 | 1.80 (-0.71, 4.30) | 0.54 (-1.96, 3.03) |
| Monitoring | 0.12 | 2.66 (-0.03, 5.36) | 0.15 (-2.35, 2.65) |
| Discipline | 0.05 | -1.00 (-2.96, 0.97) | 1.29 (-0.59, 3.18) |
| Control | 0.66 | -0.03 (-1.59, 1.53) | 0.50 (-0.95, 1.95) |
| Reinforcement | 0.09 | -1.17 (-3.16, 0.82) | 0.72 (-1.35, 2.79) |
| **Sedentary behavior**^‡^ |  |  |  |
| Limit Setting | 0.46 | 2.74 (-7.65, 13.12) | -1.39 (-11.09, 8.30) |
| Monitoring | 0.96 | -6.72 (-17.27, 3.83) | -8.73 (-21.19, 3.73) |
| Discipline | 0.53 | 1.43 (-6.93, 9.80) | -3.96 (-13.61, 5.69) |
| Control | 0.42 | 4.60 (-1.48, 10.68) | 0.29 (-6.94, 7.52) |
| Reinforcement | 0.51 | 5.02 (-3.49, 13.52) | 0.31 (-9.09, 9.71) |

*MVPA* moderate to vigorous physical activity, *p-for-int.* p-value for interaction term

*Young = 8-11 years of age, Old = 12-16 years of age

^†^Moderate to vigorous physical activity defined as ≥441 counts/15 seconds, reported in minutes/day

^‡^Sedentary behavior defined as ≤17 counts/15 seconds, reported in minutes/day

All models are survey-weighted and adjusted for the following covariates: child’s age group, child’s age group*parenting practice, parent’s age, child’s sex, parent’s sex, child’s BMI, parent’s BMI, parent’s education, household income, SOL Youth center, Hispanic/Latino background
